# Calcium Ionophore, Calcimycin, Kills* Leishmania* Promastigotes by Activating Parasite Nitric Oxide Synthase

**DOI:** 10.1155/2017/1309485

**Published:** 2017-10-18

**Authors:** Igor Grekov, António R. Pombinho, Tatyana Kobets, Petr Bartůněk, Marie Lipoldová

**Affiliations:** Institute of Molecular Genetics of the ASCR, v.v.i., Prague, Czech Republic

## Abstract

Leishmaniasis is an infectious disease caused by protozoan parasites of the genus* Leishmania*. There is no vaccine against human leishmaniasis and the treatment of the disease would benefit from a broader spectrum and a higher efficacy of leishmanicidal compounds. We analyzed the leishmanicidal activity and the mechanism of action of the calcium ionophore, calcimycin.* L. major* promastigotes were coincubated with calcimycin and the viability of the cells was assessed using resazurin assay. Calcimycin displayed dose-dependent effect with IC_50_ = 0.16 *μ*M. Analysis of propidium iodide/LDS-751 stained promastigotes revealed that lower concentrations of calcimycin had cytostatic effect and higher concentrations had cytotoxic effect. To establish the mechanism of action of calcimycin, which is known to stimulate activity of mammalian constitutive nitric oxide synthase (NOS), we coincubated* L. major* promastigotes with calcimycin and selective NOS inhibitors ARL-17477 or L-NNA. Addition of these inhibitors substantially decreased the toxicity of calcimycin to* Leishmania* promastigotes. In doing so, we demonstrated for the first time that calcimycin has a direct leishmanicidal effect on* L. major *promastigotes. Also, we showed that* Leishmania* constitutive Ca^2+^/calmodulin-dependent nitric oxide synthase is involved in the parasite cell death. These data suggest activation of* Leishmania* nitric oxide synthase as a new therapeutic approach.

## 1. Introduction

*Leishmania* is the genus of protozoan parasites which cause leishmaniasis. The reservoir hosts of most* Leishmania* species from which parasites are transmitted to humans by phlebotomine sand flies are rodents and canids (zoonotic leishmaniasis); transmission from infected to noninfected humans occurs only in some* Leishmania* species (anthroponotic leishmaniasis) (reviewed in [1]). In some cases, parasites can be transmitted from infected to noninfected humans also through needle sharing among intravenous drug users [2] and by organ transplantation and blood transfusion (reviewed in [3]). Congenital transmission from mother to child was also described [4]. There are 12 million people infected with* Leishmania* and 350 million people at risk of infection in 98 countries [5]. Recent refugee crisis caused a devastating outbreak of leishmaniasis in the Middle East and North Africa [6]. This outbreak and the global warming are the main factors promoting the spread of leishmaniasis to Europe [7–9] and to North America [10].

Despite its huge impact on the populations in vast areas, leishmaniasis is one of the most neglected diseases. Up to date, no effective vaccine against human leishmaniasis has been developed [11, 12]. The spectrum and efficacy of available antileishmanial drugs are also limited (reviewed in [13]). The first-line drugs, including amphotericin B and pentavalent antimonials, have serious side effects [13]. Thus, there is a continuing need for new targets for antileishmanial therapy and new chemical substances with leishmanicidal effect.

Calcimycin (A23187, calcium ionophore) is a carboxylic acid antibiotic isolated from* Streptomyces chartreusensis* [14]. The compound is an ionophore selective for divalent cations, particularly Ca2+, Mg2+, and Mn2+ [15, 16]. The effects of calcimycin on mammalian cells include the hyperactivation of constitutive Ca2+/calmodulin-dependent nitric oxide synthase (NOS) [17, 18], a homolog of which was found both in* Trypanosoma* [19] and in* Leishmania* [20]. Calcimycin was previously shown to decrease ^3^[H]-thymidine incorporation into* Leishmania enriettii* released from SDS-lysed macrophages stimulated by lipopolysaccharide (LPS) [21]. Moreover, pretreatment of macrophages by calcimycin before their infection with* Leishmania major* led to a decrease of ^3^[H]-thymidine incorporation by intracellular amastigotes [22]. Yet, there have been no data about the effect of calcimycin on* Leishmania* promastigotes and no data about the mechanism of action of calcimycin.

We therefore examined the antipromastigote activity of calcimycin and described its mode of action by showing that it activates parasite's nitric oxide synthase.

## 2. Materials and Methods

### 2.1. *L. major* Culture and Maintenance

*Leishmania major *LV 561 (MHOM/IL/67/LRC-L137 JERICHO II) were stored in the overlay with 10% dimethyl sulfoxide in liquid nitrogen as subculture 0. Parasites were recovered and routinely cultured for 7 days at 23°C in saline-neopeptone-blood 9 (SNB-9) medium [23]. Solid phase and overlay for SNB-9 were prepared from Bacto™ Agar (cat. number 214010, Becton, Dickinson and Company, Franklin Lakes, NJ), Bacto Neopeptone (cat. number 211681, Becton, Dickinson and Company), NaCl, and defibrinated rabbit blood (Bioveta, a. s., Ivanovice na Hané, Czech Republic). The overlay was supplemented with 50 *μ*g/ml gentamicin (cat. number G1272, Sigma, St. Louis, MO). For promastigote growth inhibition assay, subcultures #2 of* L. major* were cultivated in Schneider's Insect Medium (cat. number S0146, Sigma) supplemented with 50 *μ*g/ml gentamicin (cat. number G1272, Sigma), 63.7 *μ*g/ml penicillin G potassium salt (cat. number PENK, Sigma), 100 *μ*g/ml streptomycin sulfate salt (cat. number S6501, Sigma), 2% human urine, and 10% heat-inactivated fetal bovine serum (cat. number F2442, Sigma).

### 2.2. Promastigote Growth Inhibition Assay

*Leishmania* promastigotes in the logarithmic phase of growth were seeded into black 384-well plates (cat. number 3571, Corning, New York, NY) at a density of 15,000 parasites/25 *μ*l/well in supplemented Schneider's Insect Medium using the Multidrop Combi (Thermo Fisher Scientific, Waltham, MA). Immediately after plating* Leishmania* promastigotes, calcimycin and the reference compound amphotericin B, which is currently the best option for treatment of visceral leishmaniasis [9], were added in a range of concentrations and incubated for 48 hours at 23°C. The time of incubation was chosen as one with proven efficiency in previous experiments by others [24]. The assays were performed in triplicate. The metabolic capacity of the parasites was measured after 2.5 h coincubation with the CellTiter-Blue® Reagent (cat. number G8082, Promega, Madison, WI) using EnVision Plate Reader (PerkinElmer, Waltham, MA). The data were fitted using nonlinear regression (exponential, one-phase decay). Half maximal inhibitory concentration (IC_50_) was calculated as a concentration of the compounds, at which the viability of the parasites was 50%.

### 2.3. *Leishmania* Promastigote Counts

**Figure 1:**
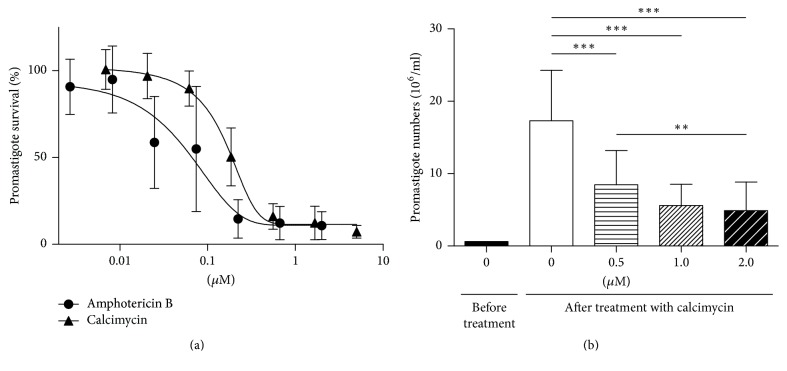
*Viability of L. major promastigotes treated with calcimycin.* (a) The percentage of viable* L. major* promastigotes in cultures treated with calcimycin and amphotericin B in comparison with untreated culture (taken as 100%). Data are presented as mean ± SD (three independent experiments; each assay was performed in triplicate). (b) Numbers of* L. major* promastigotes were counted using Z2 COULTER COUNTER. The column “0 *μ*M, before treatment” reflects starting promastigote concentration in the beginning of coincubation with calcimycin. A column labeled 0 *μ*M shows number of nontreated promastigotes after 48 hours of incubation. Columns labeled 0.5 *μ*M, 1.0 *μ*M, and 2.0 *μ*M show promastigote numbers after another 48 hours of coincubation with corresponding concentrations of calcimycin. Data are presented as mean ± SD (six independent experiments; each assay was performed in triplicate). Promastigote numbers were compared using Mann–Whitney test. *∗∗* corresponds to *P* ≤ 0.01; *∗∗∗* corresponds to *P* ≤ 0.001.

After 48-hour incubation with 0.5, 1.0, and 2.0 *μ*M calcimycin or without calcimycin,* L. major* promastigotes were counted in duplicates with Z2 COULTER COUNTER (Beckman Coulter, Inc., Brea, CA). The aforementioned concentrations of calcimycin were selected based on the results of promastigote growth inhibition assay (Figure 1(a)), representing submaximal and maximal leishmanicidal effect. For counting, 50 *μ*l of* Leishmania *parasite suspensions was diluted in 20 ml of ISOTON II Diluent (cat. number 8546719, Beckman Coulter, Inc.).

### 2.4. Analysis of Cell Death of* L. major* Promastigotes

After 48-hour incubation with 0.5, 1.0, and 2.0 *μ*M calcimycin,* L. major* promastigotes were washed and incubated with propidium iodide (PI) (cat. number P4170, Sigma) in phosphate-buffered saline (PBS) for 10 minutes in the dark at room temperature. After that, laser dye styryl- (LDS-) 751 (Invitrogen, Carlsbad, CA) was added and the samples were incubated for additional 20 minutes under the same conditions. Then the samples were washed, resuspended in PBS, and analyzed for fluorescence using flow cytometer FACSCalibur (Becton, Dickinson and Company). PI is a dye staining necrotic and late apoptotic cells with a penetrable plasma membrane. PI fluorescence was detected in both orange (FL2 channel, 585 ± 21 nm bandpass filter) and red (FL3 channel, >670 nm longpass filter) ranges of the spectrum. With our instrument settings, PI positive cells generate an average angle of 45 degrees between *y*-axis (FL2 channel) and *x*-axis (FL3 channel). LDS-751 is a cell permeable dye, which is accumulated in polarized mitochondria. Thus, LDS-751 stains nonapoptotic cells and does not stain the early and late apoptotic or necrotic cells. LDS-751 fluorescence was measured in the red (FL3 channel, >670 nm longpass filter) part of the spectrum. The samples were also analyzed by light scatter. For each sample, 10,000 events were acquired.

### 2.5. Blocking Constitutive Ca2+/Calmodulin-Dependent NOS

*L. major *promastigotes were analyzed for growth inhibition by calcimycin (0.5 *μ*M) as described in “Promastigote Growth Inhibition Assay.” The effect of blocking constitutive NOS on* Leishmania* viability was studied by simultaneously adding selective neuronal NOS inhibitors: 50 *μ*M N-[4-[2-[[(3-chlorophenyl)methyl]amino]ethyl]phenyl]-2-thiophenecarboxamide (ARL-17477) dihydrochloride (cat. number 3319, Tocris Bioscience, Bristol, UK) or 100 *μ*M N^*ω*^-Nitro-L-arginine (L-NNA) (cat. number N5501, Sigma).

### 2.6. Software

Data obtained by flow cytometry (FCM) were analyzed using FlowJo software (Tree Star, Inc., USA). The statistical analysis including the building of the graphs was performed using GraphPad Prism 5 software (GraphPad Software, USA).

## 3. Results

### 3.1. Promastigote Growth Is Inhibited by Calcimycin and Amphotericin B

The assay of metabolic capacity of parasites demonstrated that both calcimycin and the reference compound amphotericin B are highly active against* L. major* promastigotes (Figure 1(a)) with IC_50_ of 0.159 *μ*M and 0.0614 *μ*M, respectively. The direct counting by Z2 COULTER COUNTER showed that, in cultures treated with calcimycin, the number of* L. major* promastigotes was drastically decreased in comparison with untreated cultures. The original cultures were incubated for 48 hours before treatment and adjusted to the working concentration of 0.6 × 10^6^/ml. The cultures of multiplying parasites have high growth potential and after additional 48 hours of cultivation, the concentration of parasites in untreated cultures rises 28 times, to 17.32 × 10^6^/ml, whereas in cultures grown with 0.5 *μ*M, 1.0 *μ*M, and 2.0 *μ*M calcimycin, concentrations of promastigotes were dramatically lower: 8.46 × 10^6^/ml, 5.6 × 10^6^/ml, and 4.9 × 10^6^/ml, respectively (Figure 1(b)).

### 3.2. Calcimycin Has Direct Cytotoxic Effect on* Leishmania* Promastigotes

**Figure 2:**
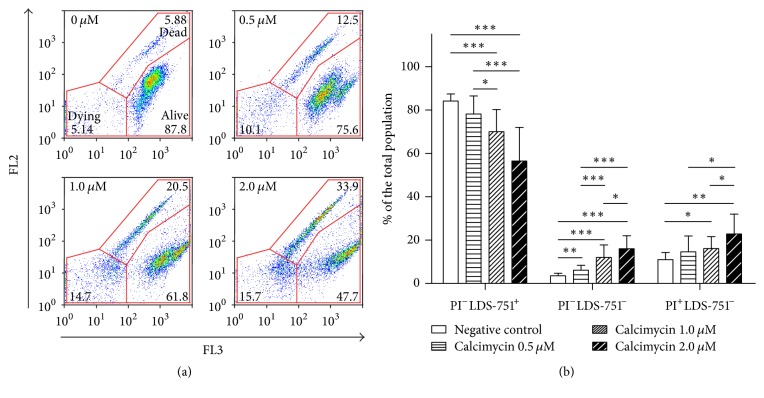
*Two-parameter, two-colour analysis of cell death in L. major promastigotes treated with calcimycin.* (a) PI stains dead cells with penetrable plasma membrane and its fluorescence was detected in both red (FL3) and orange (FL2) ranges of the spectrum. Laser dye styryl- (LDS-) 751 stains live cells with polarized mitochondria and was measured in red (FL3) fluorescence channel. Double-negative cells correspond to cells with depolarized mitochondria but an integral plasma membrane. The graphs are representative of six independent experiments. (b) The ratio of live promastigotes with intact plasma membrane and polarized mitochondria (PI^−^LDS-751^+^), cells with intact plasma membrane and depolarized mitochondria (PI^−^LDS-751^−^), and dead cells with penetrable plasma membrane and depolarized mitochondria (PI^+^LDS-751^−^) in samples treated with different concentrations of calcimycin. Data are presented as mean ± SD (six independent experiments; each assay was performed in triplicate). Percentages of particular populations were compared using Mann–Whitney test. *∗* corresponds to *P* ≤ 0.05, *∗∗* corresponds to *P* ≤ 0.01, and *∗∗∗* corresponds to *P* ≤ 0.001.

To test whether calcimycin effect was cytostatic or cytotoxic, we studied parasite staining with PI and LDS-751. Flow cytometry (FCM) analysis demonstrated that untreated samples consisted of nearly 84% of live promastigotes and 4% of dying and 11% of dead cells. At the same time, only 57% of parasites in samples treated with 2.0 *μ*M calcimycin were alive; the samples treated with 1.0 *μ*M and 0.5 *μ*M calcimycin contained 70% and 78% of live cells, respectively (Figure 2). The percentage of dead parasites with depolarized mitochondria and with the loss of plasma membrane integrity was substantially increased in cultures treated with 2.0 *μ*M of calcimycin and comprised 23%, whereas in samples treated with 1.0 *μ*M and 0.5 *μ*M calcimycin, the percentage of dead cells was 16% and 15%, respectively (Figure 2). The percentage of dying promastigotes was 16%, 12%, and 6% in cultures treated with 2.0 *μ*M, 1.0 *μ*M, and 0.5 *μ*M calcimycin, respectively. These results demonstrate direct toxicity of calcimycin to* Leishmania*.

### 3.3. Inhibitors of Constitutive NOS Block Leishmanicidal Activity of Calcimycin

**Figure 3:**
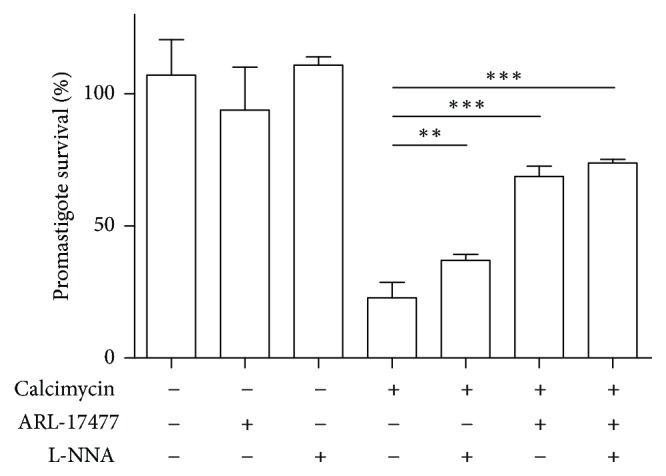
*Blocking calcimycin antipromastigote activity by ARL-17477 and L-NNA, specific inhibitors of constitutive NOS.* The metabolic capacity of* L. major* promastigotes in cultures treated with 0.5 *μ*M calcimycin and/or the specific inhibitors of constitutive NOS ARL-17477 (50 *μ*M) and L-NNA (100 *μ*M) in comparison with untreated culture (taken as 100%). Data are presented as mean ± SD (three independent experiments; each assay was performed in triplicate). Promastigote survival was compared using Mann–Whitney test. *∗∗* corresponds to *P* ≤ 0.01; *∗∗∗* corresponds to *P* ≤ 0.001.

We were able to override the leishmanicidal activity of calcimycin by adding ARL-17477 or L-NNA, specific inhibitors of constitutive Ca2^+^/calmodulin-dependent NOS. Addition of 50 *μ*M ARL-17477 or 100 *μ*M L-NNA to untreated cultures without calcimycin did not affect viability of parasites. Upon addition of 50 *μ*M ARL-17477, the numbers of viable of* L. major* promastigotes treated with calcimycin rose approximately 2 times (Figure 3). Addition of 100 *μ*M L-NNA also resulted in a significant increase in numbers of viable promastigotes.

## 4. Discussion

In the present study, we demonstrated for the first time that calcimycin has a direct dose-dependent leishmanicidal effect on* L. major *promastigotes (Figure 1). Lower doses of calcimycin inhibit* Leishmania* growth but do not augment cell death in* Leishmania* promastigotes, whereas higher doses of calcimycin cause promastigote death accompanied by the loss of mitochondrial polarization and plasma membrane integrity (Figure 2). Next, we investigated the mechanism of action of calcimycin on* L. major*. It is known that, in mammalian cells, calcimycin causes hyperactivation of constitutive Ca2^+^/calmodulin-dependent NOS [17, 18]. At the same time, NOS overexpression in mammalian cells was shown to be associated with the impairment of mitochondrial function and apoptosis [25]. As in calcimycin-treated* Leishmania*, we also observed the loss of mitochondrial potential (Figure 2); we proposed that calcimycin causes* Leishmania* apoptosis via hyperactivation of NOS. To test this hypothesis, we investigated the ability of specific inhibitors of NOS, N^*ω*^-Nitro-L-arginine (L-NNA) and ARL-17477, to attenuate the leishmanicidal effect of calcimycin. It should be mentioned that at least N^*ω*^-Nitro-L-arginine methyl ester was already shown to inhibit not only mammalian neuronal NOS [26] but also constitutive NOS of* L. amazonensis *amastigotes [20]. Indeed, our experiments demonstrated that L-NNA as well as ARL-17477 was able to block antileishmanial effect of calcimycin (Figure 3). This finding supports the hypothesis that killing of* Leishmania* parasites is mediated by hyperactivation of constitutive NOS stimulated by calcimycin.

Our results extend the data about effect of calcimycin on* Leishmania* parasites obtained by others using different experimental designs [21, 22]. In their pioneering work, Buchmuller-Rouiller and Mauel [21] infected bone marrow-derived macrophages of CBA mice with* L. enriettii*, incubated them for 4 hours with calcimycin (0.01–0.25 *μ*M) and with or without LPS, washed the cultures, and continued incubation in presence or absence of LPS for additional 20 hours. Parasite survival was measured by [^3^H]-thymidine incorporation into* L. enrietti* released from SDS-lysed macrophages. It was found that priming with calcimycin led to decrease of [^3^H]-thymidine incorporation only in presence of LPS. However, the absence of the effect of calcimycin alone might be attributed to the low concentration of calcimycin and to the very short exposure to the compound. Lanza and coworkers [22] pretreated peritoneal macrophages of BALB/c mice for 48 hours with 1 *μ*M calcimycin, washed the cells, and infected them for 3 hours with* L. major*, and [^3^H]-thymidine was added for 18 hours. This pretreatment led to a decrease of [^3^H]-thymidine incorporation by* Leishmania* amastigotes in macrophages. However, this experimental setting does not allow distinguishing if calcimycin stimulates macrophages to kill* Leishmania* or if it has cytostatic or cytotoxic effect on its own.

## 5. Conclusions

This is the first study demonstrating that calcimycin has direct cytostatic and cytotoxic effect on* Leishmania* promastigotes. We demonstrate that calcimycin-induced* Leishmania* cell death is accompanied by the loss of mitochondrial polarization and plasma membrane integrity and can be blocked by specific inhibitors of constitutive Ca2^+^/calmodulin-dependent nitric oxide synthase. The data imply that leishmanial nitric oxide synthase is crucial for the antileishmanial effect of calcimycin and suggest hyperactivation of leishmanial nitric oxide synthase as a new therapeutic approach.

## References

[1] Lipoldová M., Demant P. (2006). Genetic susceptibility to infectious disease: Lessons from mouse models of leishmaniasis. *Nature Reviews Genetics*.

[2] Cruz I., Morales M. A., Noguer I., Rodríguez A., Alvar J. (2002). Leishmania in discarded syringes from intravenous drug users. *The Lancet*.

[3] Martín-Dávila P., Fortún J., López-Vélez R. (2008). Transmission of tropical and geographically restricted infections during solid-organ transplantation. *Clinical Microbiology Reviews*.

[4] Meinecke C. K., Schottelius J., Oskam L., Fleischer B. (1999). Congenital transmission of visceral leishmaniasis (Kala Azar) from an asymptomatic mother to her child.. *Pediatrics*.

[5] Alvar J., Vélez I. D., Bern C. (2012). Leishmaniasis worldwide and global estimates of its incidence. *PLoS ONE*.

[6] Du R., Hotez P. J., Al-Salem W. S., Acosta-Serrano A. (2016). Old World Cutaneous Leishmaniasis and Refugee Crises in the Middle East and North Africa. *PLOS Neglected Tropical Diseases*.

[7] Hotez P. J. (2016). Southern Europe’s Coming Plagues: Vector-Borne Neglected Tropical Diseases. *PLOS Neglected Tropical Diseases*.

[8] Lindgren E., Naucke T., Menne B., Europe. (2004). *Climate variability and visceral leishmaniasis in Europe*.

[9] Naucke T. J., Menn B., Massberg D., Lorentz S. (2008). Sandflies and leishmaniasis in Germany. *Parasitology Research*.

[10] González C., Wang O., Strutz S. E., González-Salazar C., Sánchez-Cordero V., Sarkar S. (2010). Climate change and risk of leishmaniasis in North America: predictions from ecological niche models of vector and reservoir species. *PLOS Neglected Tropical Diseases*.

[11] Srivastava S., Shankar P., Mishra J., Singh S. (2016). Possibilities and challenges for developing a successful vaccine for leishmaniasis. *Parasites & Vectors*.

[12] Thomaz-Soccol V., Ferreira da E. S., Costa S. G., Letti L. A. J., Soccol C. R., Thomaz F. (2017). Recent advances in vaccines against *Leishmania* based on patent applications. *Recent Pat Biotechnol*.

[13] Kobets T., Grekov I., Lipoldová M. (2012). Leishmaniasis: prevention, parasite detection and treatment. *Current Medicinal Chemistry*.

[14] Reed P. W., Lardy H. A. (1972). A23187: a divalent cation ionophore.. *The Journal of Biological Chemistry*.

[15] Pressman B. C. (1976). Biological applications of ionophores. *Annual Review of Biochemistry*.

[16] Reed P. W., Lardy H. W., Mehlman M. H. H. R. W. (1972). Antibiotic A23187 as a probe for the study of calcium and magnesium function in biological systems. *The Role of Membranes in Metabolic Regulation*.

[17] Drenning J. A., Lira V. A., Simmons C. G., Soltow Q. A., Sellman J. E., Criswell D. S. (2008). Nitric oxide facilitates NFAT-dependent transcription in mouse myotubes. *American Journal of Physiology-Cell Physiology*.

[18] Werner-Felmayer G., Werner E. R., Fuchs D. (1993). Erratum: Ca2+/calmodulin-dependent nitric oxide synthase activity in the human cervix carcinoma cell line ME-180. *Biochemical Journal*.

[19] Paveto C., Pereira C., Espinosa J. (1995). The nitric oxide transduction pathway in Trypanosoma cruzi. *The Journal of Biological Chemistry*.

[20] Genestra M., Guedes-Silva D., Souza W. J. S. (2006). Nitric oxide synthase (NOS) characterization in Leishmania amazonensis axenic amastigotes. *Archives of Medical Research*.

[21] Buchmuller-Rouiller Y., Mauel J. (1991). Macrophage activation for intracellular killing as induced by calcium ionophore: Correlation with biologic and biochemical events. *The Journal of Immunology*.

[22] Lanza H., Afonso-Cardoso S. R., Silva A. G. (2004). Comparative effect of ion calcium and magnesium in the activation and infection of the murine macrophage by Leishmania major. *Biol Res*.

[23] Grekov I., Svobodová M., Nohýnková E., Lipoldová M. (2011). Preparation of highly infective Leishmania promastigotes by cultivation on SNB-9 biphasic medium. *Journal of Microbiological Methods*.

[24] Sharlow E. R., Close D., Shun T. (2009). Identification of potent chemotypes targeting *Leishmania major* using a high-throughput, low-stringency, computationally enhanced, small molecule screen. *PLOS Neglected Tropical Diseases*.

[25] De la Monte S. M., Chiche J.-D., Von dem Bussche A. (2003). Nitric oxide synthase-3 overexpression causes apoptosis and impairs neuronal mitochondrial function: Relevance to Alzheimer's-type neurodegeneration. *Laboratory Investigation*.

[26] Regli L., Held M. C., Anderson R. E., Meyer F. B. (1996). Nitric oxide synthase inhibition by L-NAME prevents brain acidosis during focal cerebral ischemia in rabbits. *Journal of Cerebral Blood Flow & Metabolism*.

[27] Grekov I. (2011). *Experimental murine leishmaniasis and its application for drug discovery and study of host-pathogen interactions [Ph.D. thesis]*.

